# Protective effect of a new generation of activated and purified bentonite in combination with yeast and phytogenic substances on mycotoxin challenge in pigs

**DOI:** 10.1371/journal.pone.0259132

**Published:** 2021-10-27

**Authors:** Pavel Horky, Hana Abigail Gruberova, Tereza Aulichova, Svetlana Malyugina, Petr Slama, Ales Pavlik, Jiri Skladanka, Misa Skoric, Sylvie Skalickova

**Affiliations:** 1 Faculty of AgriSciences, Department of Animal Nutrition and Forage Production, Mendel University in Brno, Brno, Czech Republic, European Union; 2 Faculty of AgriSciences, Department of Animal Morphology, Physiology and Genetics, Mendel University in Brno, Brno, Czech Republic, European Union; 3 Faculty of Veterinary Medicine, Department of Pathological Morphology and Parasitology, University of Veterinary and Pharmaceutical Sciences Brno, Brno, Czech Republic, European Union; University of Life Sciences in Lublin, POLAND

## Abstract

The study aimed to investigate the efficacy of new mycotoxin adsorbents based on purified and activated bentonites combined with yeast and phytogenic compounds in fattening pigs. The experiment involved 96 pigs (31.2±2.4 kg). Control (C) group was fed a diet naturally contaminated with mycotoxins (5 mg/kg deoxynivalenol, DON) without an adsorbent. Treated groups received the feed with mycotoxin adsorbents: purified and activated bentonite (T1), purified and activated bentonite, yeast derivatives, phytogenic substances (T2), and purified, activated, and sulphurated bentonite with phytogenic substances (T3). Evaluated parameters involved growth performance, organ weight, small intestine and liver histopathology, complete blood count, serum biochemistry, antioxidant status of the organism and total and free DON content in urine. In all treated groups, an significant increase in intestinal GSH and GSH/GSSG ratio was observed when compared to C. No significant effects on liver and kidney weight, complete blood count, serum or intestinal malondialdehyde concentration, or total/free DON content in urine were observed. All adsorbents improved histopathological findings in the liver when compared to C. Moreover, T1, and T2 groups showed no presence of inflammatory reaction or necrotic changes in the livers. Although, mycotoxin adsorbents investigated in this study had no significant impact on pig growth performance, they reduced the oxidative stress, and on the tissue level they protected the jejunal tissue and liver parenchyma under deoxynivalenol challenge.

## 1. Introduction

Mycotoxins are a persistent global issue and cause significant production losses in pigbreeding. Furthermore, recent climate change is responsible for the changing spectrum of mycotoxins in food and feed [1,2]. Despite improvements in agricultural and production practices, mycotoxin contamination cannot be prevented, and contaminants are virtually ubiquitous at certain concentrations in the average human and animal diet. Whereas aflatoxins can be easily removed by several adsorbents, eliminating of deoxynivalenol (DON) is still challenge.

Pigs are considered very sensitive to mycotoxins’ effects [3,4]. Depending upon the dose, frequency, and duration of exposure, DON may have either an immunostimulatory or immunosuppressive effect. Chronic exposure to low doses of mycotoxins in pigs can lead to anorexia and weight loss, and induce immunotoxicity. Acute exposure to high doses can cause diarrhoea, vomiting, leucocytosis, circulatory shock, and ultimately lead to death [5,6]. DON can elicit an inflammatory response by acting on the ribosome, inducing ribotoxic stress that activates the MAPK pathway and induces the expression of inflammation-related genes of proinflammatory cytokines [5]. The established dietary limit for growing pigs should not exceed 1 mg/kg [7–9].

The economic impact of DON contamination includes pig mortality, increased veterinary costs, and reduced meat production. As a result, there is ongoing research and development of strategies to eliminate or inactivate DON and other mycotoxin production or reduce their occurrence in feed [10]. A common approach to DON decontamination in feed is to use mineral adsorbent materials. Adsorption materials can bind mycotoxins in the gastrointestinal tract and reduce absorption and systemic toxicity [10–12]. Because adsorption is essentially a surface phenomenon, its effectiveness is affected by several physical properties such as the size and distribution of the porous material, the total charge, and its distribution.

The most significant criteria for evaluating adsorbents include the stability of the sorbent-toxin bond and their efficacy over a wide pH range, as the product is expected to function in the gastrointestinal tract [10]. The most used detoxifiers are clay minerals (aluminosilicates) composed of silicates of hydrated aluminium, iron, and magnesium, which usually contain alkali metal and alkaline earth metal ions. Clays are finely granulated earthy substances (diameter below 2 mm), which, after wetting, show certain plasticity. A mixture of tetrahedral and octahedral aluminium plates with alumina and hydroxy groups is the fundamental structural unit of silicate clay minerals [10]. This group includes two main subclasses: phyllosilicates, which include bentonites, montmorillonites, smectites, and tectosilicates, which include zeolites [10]. The advantages of mineral adsorbents are their abundant natural occurrence, low cost, and easy modification in increasing the adsorption capacity of mycotoxins [13].

However, the effectivity of clay minerals in reduce DON adsorption is limited. Despite this, DON possesses some structural features that could improve its reactivity—the epoxy ring, hydroxyl groups, and an α,β-unsaturated carbonyl group. In this context, clay minerals can be modified to increase their binding capacity [14]. Numerous studies have reported compounds with free sulphur groups as a potential DON detoxifying agent in vivo and in vitro [15–20]. Nucleophilic addition reaction of the thiol group of glutathione and DON has been demonstrated using HRMS and NMR spectrocopy. This reaction proceeds under mild conditions as a physiological pH and temperature [14]. In this modification, used additional substances with free sulphur group such as sodium metabisulphite, monome-thylamine or sodium sulphite can be used in combination other DON adsorbents [17,21,22].

More recent attention has focused on the application of yeast cell walls for DON elimination [21,23]. Main compounds identified to be perspective in term of DON adsorption are β-glucans, glucomanns and mannan-oligosaccharides [22]. Prerequisite for good adsorption capacity of yeast cell walls is a structural integrity and a non-viable cells. In particular, S. Cerevisiae have been especially tested for this purpose [24,25].

In addition, several phytoactive compounds such as essential oils, flavonoids or organic acids have been discovered to prevent cytotoxicity caused by DON [26–28]. A mechanism of action is based on protecting tissues against oxidative stress, support enzymatic detoxification and hepatoprotection [29,30]. However, their effects vary on the applied dose, the genotype and growing conditions of the plants. Thus, they do not act directly against the elimination of DON, but rather address its consequences [31].

The study´s aim was to verify the impact of purified and activated bentonite combined with yeast and phytogenic substances on fattening pigs’ growth performance and health status. In our study, mycotoxin adsorbents based on bentonite subjected to a several-step activation and purification were used. This process results in the change of the original physicochemical properties and an increase in the number of binding sites and adsorption capacity of the final product. We examined the combination with yeast and phytogenic substances for further increase its efficacy.

## 2. Materials and methods

### Animal experiment

The study was conducted according to the guidelines of the Declaration of Helsinki, and approved by the Institutional Review Board (or Ethics Committee) of Expert Commission for Ensuring the Welfare of Experimental Animals of Mendel University in Brno (protocol code 16OZ27083/2014-17214 and date of approval 20 May 2019). A total of 96 experimental pigs (castrated males DanBred) with an average weight 31.2 kg were housed in 36 identical pens measuring 2.43 x 1.46 m. The animals were exposed to the artificial lighting sources with light intensity of 45 lux in the mode 12 hour-light and 12-hour dark cycle. According to the pre-fattening pigs’ requirements, the microclimatic conditions of pigs’ housing were maintained by an artificial computer-controlled ventilation system. The basic microclimatic standards were maintained following the requirements of the Act on the protection of animals against cruelty (No. 246/1992 Coll.).

The composition and nutritional values of the feed ration are shown in Tables 1 and 2. The diet was compiled according to the Nutritional Requirements for DanBred Pigs [32].

**Table 1:** Ingredient composition of the experimental diet for pigs (%).

Ingredient	%
Wheat ^A^	50.00
Maize	10.00
Barley	10.95
Pea	4.00
Wheat bran	2.00
Wheat flour	5.00
Extracted soybean meal	8.60
Extracted rapeseed groat	5.00
Animal fat	0.50
L-Lysine HCl 98	0.26
L-Threonine 98	0.14
DL Methionine 99	0.04
L-Tryptophan 20	0.14
Calcium carbonate (ground limestone)	1.04
Feeding salt	0.47
Monocalcium phosphate	0.87
Mineral premix ^B^	1.00

^A^: In experimental groups and control group, the fungal infected wheat was used.

^B^: Provided per kg of complete diet: Vitamin A, 5000 IU; Vitamin D3, 800 IU; Vitamin E, 30 IU; Vitamin K3, 1.0 mg; Biotin, 0.05 mg; Folic acid, 0.3mg; Niacin, 10 mg; D-pantothenic acid, 10 mg; Riboflavin, 3.6 mg; Thiamine, 1.0 mg; Pyridoxin, 1.5 mg; Choline, 800 mg; Zn (ZnSO_4_), 120 mg; Fe (FeSO_4_), 125 mg; Cu (CuSO_4_.5H_2_O),15mg/kg; Mn (MnSO_4_.H_2_O), 10mg/kg; I (KI), 0.15 mg; Se (Na_2_SeO_3_), 0.2 mg.

**Table 2:** Chemical composition of the feed rations (dry matter).

Indicator	Feed rations
Metabolizable energy (MEp), MJ	13.15
Dry matter (%)	88.93
Protein (%)	16.79
Starch (%)	41.07
Fat (%)	2.52
Fiber (%)	3.41
Ash (%)	5.48
Amino acids, (g)	
Lysin	10.03
Methionine	3.26
Methionine + cysteine	6.98
Threonine	7.45
Tryptophan	3.59
Arginine	10.58
Histidine	4.64
Isoleucine	7.28
Leucine	13.29
Phenylalanine	8.47
Valine	8.70
Tyrosine	5.96

All experimental animals were provided ad libitum access to feed and drinking water. Also, the unconsumed feed mixture was monitored. In the experimental and control groups, the feed ration was composed of wheat in which high concentrations of mycotoxins were detected (Table 3). The naturally contaminated wheat was obtained from a Czech wheat producer (harvested in 2019). The presence of the toxin was detected by regular monitoring by the producer. For the study purpose, a total of 57 dietary mycotoxins were analyzed using an Acquity UPLC® System liquid chromatography (Waters, Milford, MS, USA) in conjunction with a QTRAP® tandem mass spectrometer (Applied Biosystems, Toronto, ON, Canada) according to the protocol published previously [33].

**Table 3:** The content of mycotoxins in the feed mixtures (μg/kg of dry matter).

Mycotoxin*	Content of Mycotoxins
beauvericin	5.3
deoxynivalenol	5000.0
deoxynivalenol-3-glucoside	170
enniatin A	-
enniatin A1	2.3
enniatin B	25
enniatin B1	8.7
fumonisin B1	140.0
T-2 toxin	11.0
tentoxin	5.3
zearalenone	38

* The following mycotoxins were below the detection limits: 3-acetyldeoxynivalenol; 15-acetyldeoxynivalenol; aflatoxin B1; aflatoxin B2; aflatoxin G1; aflatoxin G2; agroclavine; alternariol; alternariol-methylether; citrinin; cyclopiazonic acid; diacetoxyscirpenol; ergocornine; ergocorninine; ergocristine; ergocristinine; ergocryptine; ergocryptinine; ergometrine; ergosine; ergosinine; ergotamine; fumonisin B3; fumonisin B2; fusarenon X; gliotoxin; HT-2 toxin; meleagrin; mycophenolic acid; neosolaniol; nivalenol; ochratoxin A; patulin; paxilline; penicillic acid; penitrem A; phomopsin A; roquefortine C; sterigmatocystin; stachybotrylactam; tenuazonic acid; verruculogen; α-zearalenol; β-zearalenol.

A total of 96 pigs were included in the experiment at an average weight of 31.2 ± 2.4 kg. Animals were fed the experimental diets for 35 days and were divided into six groups of 24 animals (6 pens x 4 pigs). The control group (C) was fed a naturally contaminated wheat diet with high levels of DON without adsorbents. Other treated groups were fed with the same diet with the addition of the tested mycotoxin adsorbents. All mycotoxin adsorbents were purchased from Addicoo group, s.r.o., Czech republic. Treated group T1 was supplemented with 100% purified and activated bentonite (based on a product Fortisorb Premium, Addicoo group, s.r.o., Czech Republic) at a dose of 1.5 kg/ton. T2 group was supplemented with an adsorbent containing 75% purified and activated bentonite, 17.8% yeast cell wall derivatives, and 7.2% phytogenic substances (based on a product Fortisorb Phyto, Addicoo group, s.r.o., Czech Republic) at a dose of 2 kg/ton. T3 group was supplemented with mycotoxin adsorbent based on 95% purified, activated, and sulphurated bentonite with 5% phytogenic substances at a dose of 3.5 kg/ton.

The weight of the animals was monitored at regular weekly intervals. The average daily feed intake and feed conversion were monitored for the periods: 1–14 days; 15–28 days; 29–35 days. Six randomly selected pigs from each group ranging in final weight from 62.4 to 76.5 kg were slaughtered on the same day. The animals were left to rest and fast for about two hours before slaughtering. They were then slaughtered by electrical stunning (350 V, 4 A) and exsanguination. In each slaughtered pig, kidney and liver weight were determined as percentages of the pigs’ live weight. After slaughter, blood samples were collected via the jugular vein into EDTA tubes and 10 mL heparinized vacuum tubes, then aliquots were centrifuged at 3000 x g for 10 min to collect plasma, which was then frozen at -20°C until analysis; reduced and oxidized glutathione (GSH/GSSG), malonyldialdehyde (MDA), glutathion peroxidase (GPx) and biochemical parameters). Liver and intestine samples were collected, washed in PBS buffer and placed in 4% formaldehyde until histopathologic evaluation. Urine was collected by punction of the bladder and frozen at -20°C until total and free DON analysis. Middle jejunum (3.5 m distal from duodenum) samples were collected and frozen at -20°C until analysis (MDA).

### Scanning electron microscopy

**Fig 1:**
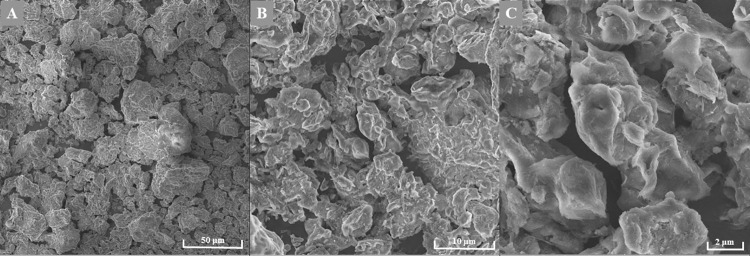
Structure of purified and activated bentonite at different magnifications (SEM). A) 1.00 kx, B) 5.00 kx and C) 15.0 kx.

For documentation of the activated bentonite structure (Fig 1), a scanning electron microscope MIRA3 LMU (Tescan, Czech Republic) was used, equipped with a high brightness Schottky field emitter for low noise imaging fast scanning rates. The SEM is fitted with an In-Beam SE detector. An accelerating voltage of 15 kV and beam currents about 1 nA gives satisfactory results regarding maximum throughput. Magnification 40 KX was used.

### Feed ration analysis

All feed samples were oven-dried at a temperature of up to 50°C, then ground with a grinder to a particle size of 1 mm and analyzed for the basic nutrient content. The fiber was determined on an A200 Fiber Analyzer (ANKOM, Czech Republic), nitrogenous substances according to the Kjeldahl method (N × 6.25), and fat (by direct extraction according to Soxhlet method) [34]. The ash was determined after burning for 4.5 hours in an oven at 550 ˚C. Energy (combustion heat, BE) was determined using a calorimeter (IKA C 5000 Werke, Germany).

### Amino Acid Analysis (AAA)

The method for AAA was adopted from Husek and Sweeley et al. Briefly, 5 g of sample was hydrolyzed according to [35]. 1 mL of hydrolysate was diluted with 2.5 mL of deionisedwater. The extracts were filtered and passed through an SCX cartridge, previously conditioned according to manufacturer protocol (UCT, Bristol, USA). The obtained solution was dried under N_2_. Each dried residue was dissolved in 60 μL water and 40 μL ethanol/pyridine (4:1). Five μL ethyl chloroformate was added to the mixture. Finally, 150 μL of chloroform was added.

Derivatized samples were analyzed using the GC system (Agilent 6890, Santa Clara, CA) equipped with an FID. Separation of compounds was conducted on a 10 m CP-Sil 19 capillary column (Agilent, USA) using nitrogen 5.0 as the carrier gas (Siad, Czech republic). The injection volume was 1 μL, and the flow rate was set at 0.7 mL/min. The injector temperature was 250°C with a split ratio of 50:1, and the FID temperature was 250°C. The oven temperature was programmed as follows: the column was held initially at 140°C, then increased to 280°C at 40°C/min and held for 3 min. Chromatographic data were recorded and integrated using Clarity software (Data Apex, Czech republic) [36].

### Analysis of urine samples on DON and DON 3-glucuronide (DON-GlcA)

Urinary DON and DON-GlcA concentrations were measured using a two-step process. Stored urine samples were allowed to thaw and were then centrifuged at 2000 g. Supernatant pH was adjusted to pH 6.8 with the dropwise addition of KOH or HCl. An aliquot (250 μL) m was digested using β-glucuronidase (20 000 U/mL) in a thermoshaker at 37°C for 18h, ensuring a gentle mixing. Digested and native urine samples were passed through IAC columns (Vicam, USA) with 2 mL of methanol. Free and total DON were analyzed by ELISA commercial kit (Elabscience, USA) according to manufacturer protocol. Creatinine was measured by a commercial kit (Elabscience, USA) according to manufacturer protocol.

### Biochemical analysis

Blood samples were collected from all experimental animals to determine haematological (haemoglobin, hematocrit, erythrocytes, leukocytes, and platelets) and biochemical parameters (albumin, aspartate transaminase (AST), gamma-glutamyltransferase (GGT), glucose, total bilirubin and protein, urea). Spectrometric analyses were performed by a Konelab T20xt biochemical analyzer (Thermo Fisher Scientific) and commercially available reagents, according to [37]. Furthermore, glutathione peroxidase (GPx) was monitored in the blood according to [38]. Colorimetric assay of determination of malonyl dialdehyde (MDA) was carried out according to the manufacturer protocol (Elabscience, USA). In brief, blood samples were defrosted and used immediately. Tissue samples (500 mg) were defrosted and homogenized in lysate buffer (0.5 mL). Aliquot was centrifuged (10000 x g for 10 minutes) and used in the assay. Twenty μL of sample or standard was put into each test tube. Reagent 1 (20 μL), reagent 2 (600 μL) and reagent 3 (200 μL) were added to the samples or standards. The mixture was incubated at 100°C for 40 min in a thermoblock (BioSan, USA). After cooling, the optical density was measured within 532 nm. A reduced (GSH), oxidized (GSSG) glutathione and their ratio GSH/GSSG was carried out according to the manufacturer protocol (SigmaAldrich, USA). Briefly, 40 μL of standard or sample was added to each well. 120 μL of buffer 1 was added and incubated in 37°C 1 hour (thermomixer shaker, BioSan, USA). 20 μL of substrate working solution, coenzyme working solution, enzyme working solution were added to each well. Optical density was measured 405 and 415 nm. The pseudo-end point method was chosen to determine the results.

For the optical density reading, the Synergy HTX Microplate reader (BioTek, USA) was used.

### Histopathology analysis

Tissue samples of the liver (taken from the right lobe of the liver, lobus hepatis dexter) and the intestine (taken from the middle jejunum, 3 cm) were collected and immediately fixed in 4% formaldehyde solution to investigate and evaluate pathomorphological changes. Fragments of tissues were cut at 3.0 μm, then positioned onto Superfrost Plus slides (Leica, UK) with the orientation core placed up on the slide. All tissue blocks were oriented the same way; then, the entire tissue block was cut with the remaining sections dipped in wax and stored at room temperature. The sections were stained with hematoxylin and eosin according to standard procedures. Pictures were taken using an inverted Olympus microscope IX 71S8F-3 (Tokyo, Japan) at the magnification 10–20 x for liver samples and 10x magnification for jejunum.

### Statistics

The data were analyzed using STATISTICA.CZ, version 12.0 (Czech Republic). The results were expressed as a mean from all samples ± standard deviation. Experiments were performed in triplicate. Statistical significance was determined by examining the basic differences among groups using ANOVA and Schaffer’s method for all parameters. The differences with p<0.05 were considered significant.

## 3. Results

### Feed efficiency during the experiment

The average scores of feed intake, conversion and daily weight gain were compared in order to evaluate feed efficiency. During all three monitored periods (1–14 days, 15–28 days, 29–35 days), no statistically significant differences in efficiency between the control and experimental groups were observed. All results of pig’s efficiency traits are presented in Table 4.

**Table 4:** Growth performance of pigs.

Feed efficiency traits	Group			
	C	T1	T2	T3
**1–14 days**				
Feed intake (kg/day)	1.64±0.08	1.62±0.11	1.63±0.07	1.70±0.12
Feed conversion (kg/kg)	1.99±0.07	1.94±0.06	1.97±0.08	1.94±0.09
Weight gain (kg/day)	0.82±0.04	0.83±0.04	0.83±0.03	0.87±0.06
**15–28 days**				
Feed intake (kg/day)	2.25±0.14	2.17±0.14	2.14±0.11	2.45±0.16
Feed conversion (kg/kg)	2.12±0.10	2.06±0.07	2.05±0.06	2.08±0.14
Weight gain (kg/day)	1.01±0.07	1.06±0.08	1.04±0.05	1.03±0.07
**29–35 days**				
Feed intake (kg/day)	2.41±0.12	2.37±0.14	2.41±0.16	2.47±0.16
Feed conversion (kg/kg)	2.56±0.17	2.54±0.11	2.53±0.11	2.48±0.19
Weight gain (kg/day)	0.94±0.05	0.94±0.06	0.95±0.05	0.99±0.10
**Entire experiment**				
Feed intake (kg/day)	1.99±0.08	1.99±0.09	1.98±0.07	2.09±0.12
Feed conversion (kg/kg)	2.17±0.05	2.11±0.05	2.12±0.04	2.11±0.08
Weight gain (kg/day)	0.92±0.03	0.94±0.05	0.94±0.03	0.96±0.04

### Body and organ weights of pigs

During dissection, the liver and kidneys were removed (Table 5). The weight of individual organs was related to the overall weight of the carcass. No statistically significant differences were observed between the control and experimental groups. The body weight was not significantly affected during the experiment (Table 6).

**Table 5:** Liver and kidneys percentage (%) of body weight.

	Group				
	C	T1	T2	T3	p-value
Liver (%)	2.01±0.13	2.23±0.19	2.03±0.13	1.91±0.32	0.122
Kidneys (%)	0.39±0.04	0.40±0.06	0.40±0.04	0.36±0.06	0.385

**Table 6:** Body weight of pigs´ during experiment.

Time period (kg)	Group				
	C	T1	T2	T3	p-value
0 day	31.1±1.9	31.0±3.0	31.2±2.3	31.6±2.1	0.826
14 day	42.6±2.7	42.7±3.7	42.7±3.0	43.8±3.4	0.514
28 day	56.7±3.7	57.5±4.6	57.3±3.8	58.3±4.2	0.624
35 day	63.6±3.9	64.0±4.7	63.9±3.7	65.2±4.6	0.463

### Evaluation of antioxidant status in pigs

The first set of analyses examined the impact of mycotoxins (C group) on the antioxidant status of pigs and adsorbent ability (T1 –T3 groups) to reverse this effect. The results are present in Table 7. There was a significant difference (p <0.05) in reduced glutathione levels (GSH) between C and T1—T3. The highest GSH level was observed in the T1 group (13.6 μmol/L). No significant differences in the oxidized form of glutathione (GSSG) were found between examined groups. The GSH/GSSG ratio was significantly increased in groups T2, T3 (by 1.46 and 1.61, respectively) in comparison with C (p <0.05). The MDA level in the jejunal mucosa and in the blood was not significantly affected in any experimental groups. No significant differences were observed for the antioxidant enzyme glutathione peroxidase (GPx).

**Table 7:** Antioxidant status parameters in blood (GSH, GSSG, GSH/GSSG, MDA, GPx) and jejunal mucosa (MDA).

	Group				
	C	T1	T2	T3	p-value
GSH (μmol/L)	4.97±0.99 ^a^	13.64±3.54 ^b^	11.65±2.36 ^b^	9.82±1.96 ^b^	0.001
GSSG (μmol/L)	9.99±4.36	11.46±3.85	9.04±3.24	7.38±4.38	0.512
GSH/GSSG	0.57±0.23 ^a^	1.25±0.53 ^ab^	1.47±0.36 ^b^	1.62±0.69 ^b^	0.011
MDA intestine (nmol/mg)	45.7±3.4	46.3±2.2	48.3±4.9	47.3±1.9	0.242
MDA blood (nmol/mL)	4.35±0.54	4.33±0.74	3.90±0.61	3.52±0.83	0.394
GPx (μkat/L)	393±119	337±74	383±49	319±46	0.484

^ab^ Means within a row lacking a common superscript differ (p < 0.05).

### Haematological parameters

All haematological parameters were within the physiological range, and no significant differences indicating a health problem were observed between groups. (Table 8).

**Table 8:** Haematological parameters.

	Group				
	C	T1	T2	T3	p-value
Hemoglobin (g/L)	137±18	142±11	137±14	131±6	0.476
Leukocytes (g/L)	12.83±4.88	11.03±5.50	10.73±4.55	14.65±6.87	0.779
Hematocrit (L/L)	0.36±0.04	0.37±0.02	0.37±0.03	0.37±0.01	0.532
Platelets (g/L)	438±80	455±55	429±63	422±57	0.784
Erytrocytes (t/L)	6.15±0.64	6.36±0.27	6.20±0.69	6.12±0.21	0.456

### Biochemical parameters

To assess health status and liver function, the selected enzymes’ activities were compared between C group and treated groups (T1, T2 and T3). No significant differences were found in all studied parameters; albumin, AST, GGT, CREA, Urea, GLUC and SDH (Table 9).

**Table 9:** Biochemical parameters.

	Group				
	C	T1	T2	T3	p-value
Albumin (g/L)	29.8±2.8	30.2±3.1	26.2±3.1	30.4±2.7	0.948
AST (μkat/L)	0.89±0.32	0.58±0.04	0.63±0.27	0.57±0.18	0.355
GGT (μkat/L)	0.50±0.05	0.39±0.12	0.44±0.14	0.35±0.07	0.386
Creatinine (μmol/L)	110±13	109±13	104±13	124±4	0.117
Urea (mmol/L)	4.79±1.60	3.52±0.89	4.90±1.60	5.02±1.10	0.221
Glucose (mmol/L)	4.68±0.45	4.83±0.68	4.56±0.36	4.94±0.46	0.689
SDH (nmol/L)	0.016±0.002	0.017±0.002	0.016±0.002	0.017±0.001	0.948

### DON levels in urine

The average levels of unmetabolized free DON and total DON after enzymatic cleavage were evaluated in the urine. No significant differences between C and T1, T2 and T3 were observed. However, all treated groups showed a slightly decreased level of free and total DON compared to C (Table 10).

**Table 10:** Total and free DON levels in urine.

	Group				
	C	T1	T2	T3	p-value
Free DON (ng/mL)	38.5±28.6	28.2±8.9	25.6±13.4	30.4±12.8	0.051
Total DON (ng/mL)	44.4±31.0	37.9±19.6	32.8±14.0	40.4±13.4	0.424

### Histopathological examination of small intestine and liver

**Fig 2:**
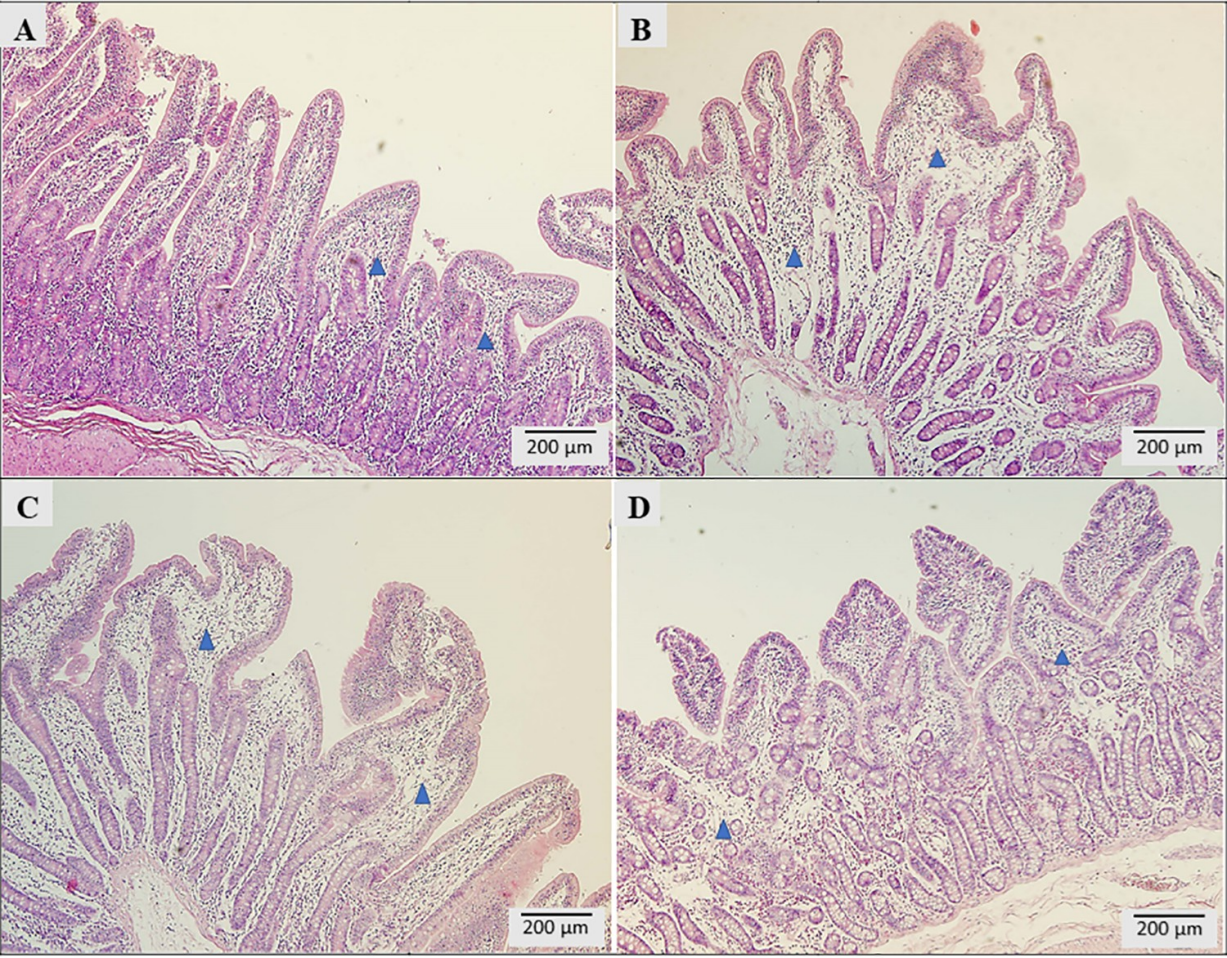
Morpho-pathological analysis of the middle jejunum. A) C group, B) T1 group, C) T2 group, D) T3 group.

Pathological changes were observed in all samples of animals in the experimental groups, with various intensities. For histopathological evaluation of the intestine, the samples were taken from the middle jejunum. The C group had predominantly marked lymphoplasmacytic inflammatory infiltrate in the mucosa with abundant eosinophils; occasional macrophages; focal hyperemia; and predominantly mild, moderately focal edema of lamina propria. Moderate lymphoplasmacytic infiltrate, lymphangiectasia of some villi, shortening and fusion of some villi, and submucosal edema have been observed (Fig 2A). The mean goblet cell count was 31/HPF. In group T1 (Fig 2B), mostly moderate lymphoplasmacytic infiltrate in the lamina propria of the mucosa was observed. Isolated, the abundant presence of eosinophils, mild, diffuse, focal edema of the lamina propria, hyperemia, focal lymphangiectasia of some villi were noted, and the mean goblet cell count was 37/HPF. Group T2 (Fig 2C) had predominantly marked lymphoplasmacytic inflammatory infiltrate in the mucosa, isolated, abundant presence of eosinophils, mild to moderate edema of the lamina propria and submucosal layer. There were abbreviated foci and villi fusions. The mean goblet cell count was 24/HPF. Group T3 (Fig 2D) had predominantly moderate lymphoplasmacytic inflammatory infiltrate with abundant eosinophils; hyperemia; mild, moderately focal lamina propria; and submucosal edema. The mean goblet cell count was 43/HPF.

**Fig 3:**
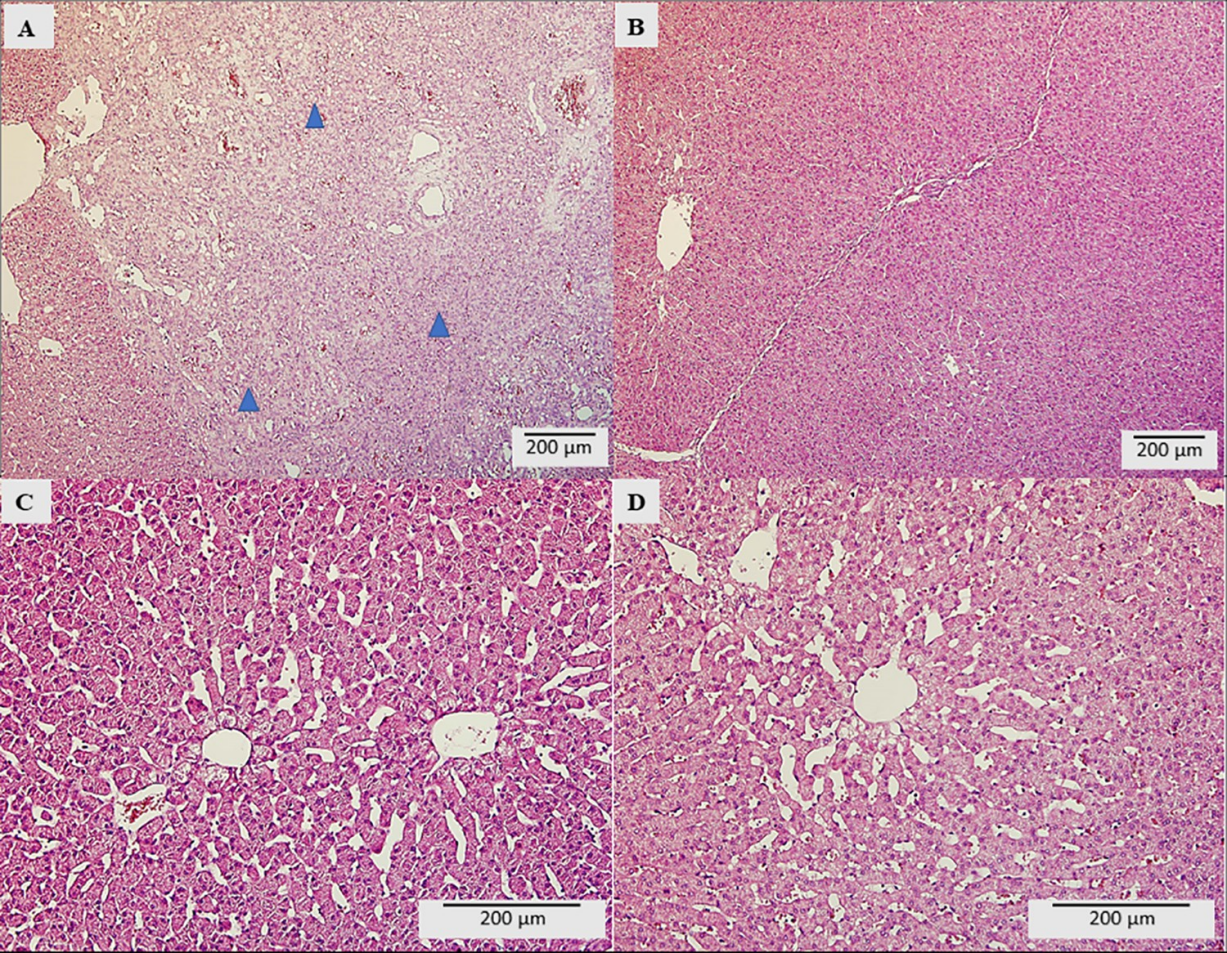
Morpho-pathological liver analysis. A) C group, B) T1 group, C) T2 group, D) T3 group.

Histopathological evaluation of the liver showed focal hepatocyte necrosis and focal proliferation of fibrovascular granulation tissue in the C group (Fig 3A). Significant focal dystrophic changes consistent with granular and hydropic degeneration of hepatocytes with a different distribution (multifocal) in the parenchyma were observed. Furthermore, moderate chronic hepatitis was found. In the T1 group, moderately significant focal dystrophic changes consistent with granular and hydropic degeneration of hepatocytes with different distribution in the parenchyma were observed (Fig 3B). Group T2 showed only slightly dystrophic focal changes in the form of granular and hydropic degeneration of hepatocytes with different distribution in the parenchyma (Fig 3C). In both groups (T1, T2), there was no presence of inflammatory reaction or necrotic changes found. The T3 group of pigs had mild dystrophic focal changes consistent with granular and hydropic degeneration of hepatocytes with different distribution in the parenchyma. In addition, smaller foci of nonspecific inflammatory response formed by the round cell infiltrate were observed (Fig 3D). The cellular infiltrates consisted mainly of lymphocytes and plasma cells.

## 4. Discussion

The main objective of the study was to evaluate the effectivity of purified and activated bentonite adsorbents enriched with phytoactive compounds and yeast cell walls on pigs´ health status and fattening performance under higher DON occurrence in feed. Three mycotoxin adsorbents (T1, T2 and T3) were tested and compared to the control group fed with contaminated feed.

T1 group was supplemented with an adsorbent based on purified and activated bentonite. However, bentonites have been reported to show weaker binding capacity for DON [39], as they have demonstrated the ability to protect minks’ skin against DON effects [40]. In vitro investigation of activated bentonite and yeast cell walls extract on intestinal epithelial barrier model showed that the yeast cell wall and bentonite clay prevented DON-induced disruption of the porcine intestinal barrier; however, each dose of mycotoxin detoxifier itself did not affect intestinal barrier disruption. Moreover, activated bentonite alone showed the ability to protect model tissue against inflammatory response as well as positive modulation of chemokine induction by MAPK signals in the highest doses (5%), whereas the best results were obtained with the combination of yeast cell walls [41]. Another study showed similar anti-inflammatory effect of activated clay minerals on the cellular level by regulation of MAPK signal pathway, however in our study we have not observed any significant changes in pigs´ growth promotion [42].

T2 group was supplemented with an adsorbent containing purified and activated bentonite, yeast derivatives, and phytogenic substances, which help the body to cope with some of the toxic effects of DON [26]. Yeast walls are known to adsorb DON at varying levels[43]. Holand et al., studied the effect of postbiotic yeast cell on performance in weaned piglets. They found a significant reduction in daily feed intake and average daily weight gain in piglets (feed mixture contained 2000 μg/kg DON without adsorbents). Fortified yeast cell wall extract maintained the performance of pigs during the tested period [44]. Similar studies have shown positive effects on pigs´ growth performance [44–46]. Yeasts are often combined with phytogenic substances in vivo to study the elimination effect of DON and other mycotoxins. The phytoactive substances themselves are further studied in vitro, where their anti-inflammatory effect has been demonstrated. However, no study has been reported across the literature to investigate the effects of phytogenic agents on ADG and ADFI in vivo.

T3 group was supplemented with mycotoxin adsorbent based on purified, activated, and sulphurated bentonite with phytogenic substances. In pig nutrition, it has been observed that the enrichment of bentonites by sulphur compounds such as sodium metabisulfite or sodium sulphite have shown positive ability to mitigate DON effects on pigs [47]. Tran, et al recently published that sodium sulphite reduced DON level (5.36 mg/kg) from contaminated maize by 75%. Pigs were fed with preserved corn at the concentration of 5g sodium sulphide/1 kg of maize. Although treatment of corn decreased levels of DON, the health parameters of the pigs did not consistently reflect this decreased exposure [48]. In the subsequent experiment Tran, et al proved that sodium sulphite did not affect liver function, redox status and blood cell counts [49]. In another study, sodium sulphite (5 g/kg with 15% of propionic acid) helped to maintain feed intake and growth performance of pigs after DON exposure in feed (44.45 mg/kg maize) [21]. The main disadvantage of sodium sulphite usage might be less efficiency to zearalenone elimination. Similarly perspective results have been obtained by sodium metabisulfite in vivo and in vitro [17,47,50].

In our study, DON concentration in contaminated feed mixture was 5 mg/kg of dry matter, which is the concentration that occurs very frequently in feed contaminated by moulds [51]. Other mycotoxins included in the diet did not exceed the established limits [52]. It has been described that this concentration causes a decrease in growth and performance, especially in young pigs, which was not significantly reflected in our experiment. In this context, Nguyen-Ba noted that the age and weight of pigs could affect perturbation and resilience on DON in feed mixture [53]. Our results are consistent with the study published by Liao et al. They did not observe significant differences of ADG and ADFI of pigs (initial body weight  =  16.3 ± 1.5 kg) between control group and treated group by DON at the doses 3 and 6 mg/kg [54]. However, significant differences between control and treated group of ADG and ADFI of 6- week-old growing pigs with an initial average BW of ~19 kg under DON challenge have been confirmed in growing pigs which are more sensitive to DON exposure [55].

It has been observed that DON exposure could affect hematologic results and the antioxidant status of pigs. The proposed mechanism of action is via activation of several mitogen-activated protein kinases (MAPKs) responsible for cell apoptosis, inflammatory response and oxidative stress [56]. However, we did not observe any significant changes in the hematologic profile of treated (T1, T2 and T3 groups) or untreated (C group) pigs. According to some studies, the main cellular targets of DON are leukocytes [57]. Surprisingly, no significant differences in leukocyte counts have been found in our study. Regarding the antioxidant status of pig´s organisms, significant increases in GSH concentration and GSH/GSSG ratio were observed in treated groups compared to control. Similar to our results, in the study by Thanh et al., DON in feed mixture (at a dose of 3100 μg/kg) did not affect growth performance, average daily gain, average daily feed intake, nor feed efficiency. However, they found partially induced oxidative stress in weaned pigs resulting in increased MDA content in the plasma and superoxide dismutase (SOD) activity in the liver. Nonetheless, no change in the activity of other antioxidant enzymes or GSH concentrations was observed in plasma and livers of piglets fed the DON contaminated diet [58]. Other studies also document increased oxidative stress (higher MDA production and reduced GSH content) in connection with the occurrence of mycotoxins in the body [59–61].

Animal studies demonstrated that exposure to DON could be measured in urine. Experimental evidence indicated unmetabolized DON, and its conjugates with glucuronic acid (15-glucuronide, DON 3-glucuronide) and glucuronidation products in urine samples in pigs [62–65]. In our study, the highest level of free and total DON was observed in the C group when compared to T1, T2, and T3 groups. Determined C group DON levels agree with the findings of other studies, in which the DON level was observed in similar levels (30 ng/mg creatinin) of total DON in pig´s urine. Daily intake was 116.84 ±217 μg/kg [62,66]. However, levels of DON in the urine change dynamically based on water and feed intake. Therefore, longer-term monitoring would demonstrate greater changes in treatment efficacy in further experiments.

Some studies investigating DON effects on organisms have reported histopathological changes of the gastrointestinal tract such as inflammatory infiltration, necrotic changes in the intestinal villi, edema of lamina propria, a decrease in the number of goblet cells in the jejunum and the ileum, intensification of apoptosis and degeneration of lymphoid cells in the gastrointestinal tract [67,68]. Considering the findings of inflammation of the intestinal mucosa with the presence of eosinophils, it should be stated that a non-specific inflammatory reaction did occur, probably arising as a result of an exaggerated reaction of the immune system. We have noticed that more significant morphopathological changes in the small intestine, such as focal necrosis, were observed. Apparently, the dose of DON has a direct effect on the morphological formation of the intestinal mucosa. These findings were partially reversed by the test substances in the treated groups (T1, T2, T3). In comparison to another study, epithelial lesions (multifocal atrophy, villi fusion, apical villi necrosis, enterocyte vacuolation, and lamina propria edema) have also been found in the intestines of pigs fed with DON contaminated diets [69]. In our experiment, similar morphopathological changes were detected in the C group and, to a lesser extent, in the T1 and T2 groups. Furthermore, similar to our study, a reduced height of villi in the small intestine was observed [45,70].

The liver, the main detoxifying organ, is strongly exposed to toxic and foreign substances. In our study, the most considerable morphological changes of liver influenced by treatment (T1, T2 and T3 groups) were observed compared to C group. C group showed, in addition to degenerative changes in the liver parenchyma, the presence of necrotic deposits, inflammatory reactions and a reparative process in the localization of these lesions. The occurrence of regressive lesions in the liver, i.e., parenchymatous and vacuolar degeneration of individual hepatocytes was confirmed by detailed histopathological study after DON administration to pigs at the dose ranged from 0.2 to 0.4 mg/kg/BW per day [71]. Similar damage has been observed in another study at the doses of DON 12 μg/kg BW per day [72]. Only reversible dystrophic changes of hepatocytes (mild to moderate intensity) were detected in all treated groups. However, no necrotic foci, fibrovascular tissue proliferation, or marked foci of inflammatory response were found in treated groups T1, T2 and T3. Detected pathomorphological changes in liver parenchyma of experimental groups refer to preventive effect of tested adsorbents in relation to development of hepatocytes necrosis and marked inflammatory reaction in parenchyma. Persisting dystrophic changes of hepatocytes in experimental groups is a reversible process.

## 5. Conclusions

This study set out to determine the influence of three groups of mycotoxin adsorbents based on purified and activated bentonites and/or sulphurated or with the addition of yeast cell derivates, phytoactive compounds a on pig health status and performance under deoxynivalenol challenge. In our study, we did not observe significant changes of pigs’ growth performance within either haematological and biochemical parameters. Slightly upward trends have been observed in GSH level and GSH/GSSG ratio in the treated groups compared to C group. The most visible changes were observed between C and treated groups in histopathological examination, which showed that the combination of activated, sulphonated bentonites, yeast extract and phytoactive compounds had a protective and improving effect at the tissue level.

## References

[1] Nesic K, Milicevic D, Nesic V, Ivanovic S. Mycotoxins as one of the foodborne risks most susceptible to climatic change. In: Nastasijevic I, Fries R, Avery S, editors. 58th International Meat Industry Conference. Procedia Food Science. 5. Amsterdam: Elsevier Science Bv; 2015. p. 207–10.

[2] Horky P, Skladanka J, Nevrkla P, Slama P. EFFECT OF DIET SUPPLEMENTED WITH ANTIOXIDANTS (SELENIUM, COPPER, VITAMINS E AND C) ON ANTIOXIDANT STATUS AND EJACULATE QUALITY OF BREEDING BOARS. Annals of Animal Science. 2016;16(2):521–32. doi: 10.1515/aoas-2015-0085 WOS:000375606300016.

[3] Andretta I, Kipper da Silva M, Lehnen C, Hauschild L, Vale M, Lovatto PA. Meta-analytical study of productive and nutritional interactions of mycotoxins in growing pigs. Animal: an international journal of animal bioscience. 2012;6:1476–82. doi: 10.1017/S1751731111002278 23031521

[4] Kipper M, Andretta I, Ribeiro AML, Pires PGD, Franceschina CS, Cardinal KM, et al. Assessing the implications of mycotoxins on productive efficiency of broilers and growing pigs. Sci Agric. 2020;77(3). e20180236 doi: 10.1590/1678-992x-2018-0236 WOS:000484594500001.

[5] Pierron A, Alassane-Kpembi I, Oswald IP. Impact of mycotoxin on immune response and consequences for pig health. Animal Nutrition. 2016;2(2):63–8. doi: 10.1016/j.aninu.2016.03.001 29767037 PMC5941016

[6] Lessard M, Savard C, Deschene K, Lauzon K, Pinilla VA, Gagnon CA, et al. Impact of deoxynivalenol (DON) contaminated feed on intestinal integrity and immune response in swine. Food and Chemical Toxicology. 2015;80:7–16. doi: 10.1016/j.fct.2015.02.013 25701311

[7] COMMISSION RECOMMENDATION on the presence of deoxynivalenol, zearalenone, ochratoxin A, T-2 and HT-2 and fumonisins in products intended for animal feeding. Official Journal of the European Union. 2006:1111–3.

[8] Holanda DM, Kim SW. Efficacy of Mycotoxin Detoxifiers on Health and Growth of Newly-Weaned Pigs under Chronic Dietary Challenge of Deoxynivalenol. Toxins (Basel). 2020;12(5):311. doi: 10.3390/toxins12050311 . 32397551 PMC7290511

[9] Horky P, Skalickova S, Urbankova L, Baholet D, Kociova S, Bytesnikova Z, et al. Zincphosphate-based nanoparticles as a novel antibacterial agent: in vivo study on rats after dietary exposure. Journal of Animal Science and Biotechnology. 2019;10. 17 doi: 10.1186/s40104-019-0319-8 WOS:000458771600001. 30805185 PMC6373129

[10] Gregorio M, Neef D, Jager A, Corassin C, Carão Á, Albuquerque R, et al. Mineral adsorbents for prevention of mycotoxins in animal feeds. Toxin Reviews. 2014;33:11. doi: 10.3109/15569543.2014.905604

[11] Awad WA, Ghareeb K, Böhm J, Zentek J. Decontamination and detoxification strategies for the Fusarium mycotoxin deoxynivalenol in animal feed and the effectiveness of microbial biodegradation. Food Additives & Contaminants: Part A. 2010;27(4):510–20. doi: 10.1080/19440040903571747 20234966

[12] Kociova S, Dolezelikova K, Horky P, Skalickova S, Baholet D, Bozdechova L, et al. Zinc phosphate-based nanoparticles as alternatives to zinc oxide in diet of weaned piglets. Journal of Animal Science and Biotechnology. 2020;11(1). 59 doi: 10.1186/s40104-020-00458-x WOS:000540838100001. 32528676 PMC7282173

[13] Elliott CT, Connolly L, Kolawole O. Potential adverse effects on animal health and performance caused by the addition of mineral adsorbents to feeds to reduce mycotoxin exposure. Mycotoxin Research. 2020;36(1):115–26. doi: 10.1007/s12550-019-00375-7 31515765 PMC6971152

[14] Stanic A, Uhlig S, Solhaug A, Rise F, Wilkins AL, Miles CO. Nucleophilic Addition of Thiols to Deoxynivalenol. Journal of Agricultural and Food Chemistry. 2015;63(34):7556–66. doi: 10.1021/acs.jafc.5b02864 WOS:000360866500012. 26242781

[15] Tran AT, Kluess J, Berk A, Paulick M, Frahm J, Schatzmayr D, et al. Detoxification of Fusarium-contaminated maize with sodium sulphite—in vivo efficacy with special emphasis on mycotoxin residues and piglet health. Arch Anim Nutr. 2018;72(1):58–75. doi: 10.1080/1745039X.2017.1418047 WOS:000429416600004. 29313386

[16] Schwartz-Zimmermann HE, Paulick M, Danicke S, Schatzmayr D, Berthiller F. Determination of deoxynivalenol sulphonates in cereal samples: method development, validation and application. World Mycotoxin J. 2014;7(3):233–45. doi: 10.3920/wmj2013.1684 WOS:000339460900001.

[17] Schwartz-Zimmermann HE, Wiesenberger G, Unbekannt C, Hessenberger S, Schatzmayr D, Berthiller F. Reaction of (conjugated) deoxynivalenol with sulphur reagents—novel metabolites, toxicity and application. World Mycotoxin J. 2014;7(2):187–97. doi: 10.3920/wmj2013.1632 WOS:000334568500009.

[18] Schwartz HE, Hametner C, Slavik V, Greitbauer O, Bichl G, Kunz-Vekiru E, et al. Characterization of Three Deoxynivalenol Sulfonates Formed by Reaction of Deoxynivalenol with Sulfur Reagents. Journal of Agricultural and Food Chemistry. 2013;61(37):8941–8. doi: 10.1021/jf403438b WOS:000330096300028. 23964860

[19] Rempe I, Kersten S, Valenta H, Danicke S. Hydrothermal treatment of naturally contaminated maize in the presence of sodium metabisulfite, methylamine and calcium hydroxide; effects on the concentration of zearalenone and deoxynivalenol. Mycotoxin Research. 2013;29(3):169–75. doi: 10.1007/s12550-013-0166-y WOS:000209500500006. 23536360

[20] Lake J, Browers M, Yin XS, Speers RA. Use of sodium bisulfite as a method to reduce DON levels in barley during malting. Journal of the American Society of Brewing Chemists. 2007;65(3):172–6. doi: 10.1094/asbcj-2007-0612-01 WOS:000248523500008.

[21] Bahrenthien L, Kluess J, Berk A, Kersten S, Saltzmann J, Huether L, et al. Detoxifying deoxynivalenol (DON)-contaminated feedstuff: consequences of sodium sulphite (SoS) treatment on performance and blood parameters in fattening pigs. Mycotoxin Research. 2020;36(2):213–23. doi: 10.1007/s12550-019-00385-5 WOS:000528385200009. 31960350 PMC7182618

[22] Tran A-T, Kluess J, Kersten S, Berk A, Paulick M, Schatzmayr D, et al. Sodium sulfite (SoS) as decontamination strategy forFusarium-toxin contaminated maize and its impact on immunological traits in pigs challenged with lipopolysaccharide (LPS). Mycotoxin Research. 2020;36(4):429–42. doi: 10.1007/s12550-020-00403-x WOS:000568751000001. 32902833 PMC7536171

[23] Weaver AC, See MT, Kim SW. Protective Effect of Two Yeast Based Feed Additives on Pigs Chronically Exposed to Deoxynivalenol and Zearalenone. Toxins (Basel). 2014;6(12):3336–53. doi: 10.3390/toxins6123336 WOS:000346794900009. 25533517 PMC4280538

[24] Chlebicz A, Slizewska K. In Vitro Detoxification of Aflatoxin B-1, Deoxynivalenol, Fumonisins, T-2 Toxin and Zearalenone by Probiotic Bacteria from Genus Lactobacillus and Saccharomyces cerevisiae Yeast. Probiotics and Antimicrobial Proteins. 2020;12(1):289–301. doi: 10.1007/s12602-018-9512-x WOS:000519537600031. 30721525 PMC7072052

[25] van der Peet-Schwering CMC, Jansman AJM, Smidt H, Yoon I. Effects of yeast culture on performance, gut integrity, and blood cell composition of weanling pigs. Journal of Animal Science. 2007;85(11):3099–109. doi: 10.2527/jas.2007-0110 WOS:000250648400036. 17609465

[26] Abdel-Wahhab MA, El-Nekeety AA, Salman AS, Abdel-Aziem SH, Mehaya FM, Hassan NS. Protective capabilities of silymarin and inulin nanoparticles against hepatic oxidative stress, genotoxicity and cytotoxicity of Deoxynivalenol in rats. Toxicon. 2018;142:1–13. doi: 10.1016/j.toxicon.2017.12.045 WOS:000425201000002. 29248467

[27] Wan MLY, Turner PC, Co VA, Wang MF, Amiri KMA, El-Nezami H. Schisandrin A protects intestinal epithelial cells from deoxynivalenol-induced cytotoxicity, oxidative damage and inflammation. Scientific Reports. 2019;9. 19173 doi: 10.1038/s41598-019-55821-4 WOS:318441237900002. PMC6915730 31844123

[28] Perczak A, Jus K, Gwiazdowska D, Marchwnska K, Waskiewicz A. The Efficiency of Deoxynivalenol Degradation by Essential Oils under In Vitro Conditions. Foods. 2019;8(9). 403 doi: 10.3390/foods8090403 WOS:000487655600029. 31514336 PMC6769570

[29] Poppenberger B, Berthiller F, Lucyshyn D, Sieberer T, Schuhmacher R, Krska R, et al. Detoxification of the Fusarium mycotoxin deoxynivalenol by a UDP-glucosyltransferase from Arabidopsis thaliana. Journal of Biological Chemistry. 2003;278(48):47905–14. doi: 10.1074/jbc.M307552200 WOS:000186731400068. 12970342

[30] Egresi A, Sule K, Szentmihalyi K, Blazovics A, Feher E, Hagymasi K, et al. Impact of milk thistle (Silybum marianum) on the mycotoxin caused redox-homeostasis imbalance of ducks liver. Toxicon. 2020;187:181–7. doi: 10.1016/j.toxicon.2020.09.002 WOS:000582386000023. 32920016

[31] Jin L, Wang W, Degroote J, Van Noten N, Yan H, Majdeddin M, et al. Mycotoxin binder improves growth rate in piglets associated with reduction of toll-like receptor-4 and increase of tight junction protein gene expression in gut mucosa. Journal of Animal Science and Biotechnology. 2017;8. doi: 10.1186/s40104-017-0210-4 WOS:000414420300002. 29118977 PMC5664444

[32] International D. Available from: https://docplayer.net/45981320-Nutritional-requirements-for-danbred-pigs.html.

[33] Chrpova J, Sip V, Sumikova T, Salava J, Palicova J, Stockova L, et al. Occurrence of Fusarium species and mycotoxins in wheat grain collected in the Czech Republic. World Mycotoxin J. 2016;9(2):317–27. doi: 10.3920/wmj2015.1917 WOS:000371816400016.

[34] AOAC. Official methods of analysis. Association of official Annalytical Chemists: Gaithersburg, MD. 2007.

[35] Zhong HY, Marcus SL, Li L. Microwave-assisted acid hydrolysis of proteins combined with liquid chromatography MALDI MS/MS for protein identification. Journal of the American Society for Mass Spectrometry. 2005;16(4):471–81. doi: 10.1016/j.jasms.2004.12.017 WOS:000228021400007. 15792716

[36] Husek P, Sweeley CC. GAS-CHROMATOGRAPHIC SEPARATION OF PROTEIN AMINO-ACIDS IN 4 MINUTES. HRC-J High Resolut Chromatogr. 1991;14(11):751–3. WOS:A1991HH35900008.

[37] Pavlik A, Slama P, Bures D, Kotrba R. EFFECT OF FEEDING ON GROWTH AND BLOOD BIOCHEMISTRY OF MALE FALLOW DEER. J Microbiol Biotechnol Food Sci. 2018;8(3):911–3. doi: 10.15414/jmbfs.2018-19.8.3.911–913 WOS:000455221000011.

[38] Urbankova L, Horky P, Skladanka J, Pribilova M, Smolikova V, Nevrkla P, et al. Antioxidant status of rats’ blood and liver affected by sodium selenite and selenium nanoparticles. Peerj. 2018;6. e4862 doi: 10.7717/peerj.4862 WOS:000434233100008. 29868274 PMC5978387

[39] Kong C, Shin SY, Kim BG. Evaluation of mycotoxin sequestering agents for aflatoxin and deoxynivalenol: an in vitro approach. Springerplus. 2014;3. 346 doi: 10.1186/2193-1801-3-346. WOS:000359054000004. PMC4101124 25045616

[40] Taszkun I, Tomaszewska E, Dobrowolski P, Zmuda A, Sitkowski W, Muszynski S. Evaluation of Collagen and Elastin Content in Skin of Multiparous Minks Receiving Feed Contaminated with Deoxynivalenol (DON, vomitoxin) with or without Bentonite Supplementation. Animals. 2019;9(12). 1081 doi: 10.3390/ani9121081 WOS:000506636400080. 31817218 PMC6940999

[41] Park S-H, Kim J, Kim D, Moon Y. Mycotoxin detoxifiers detoxifiers attenuate deoxynivalenol-induced pro-inflammatory barrier insult in porcine enterocytes as an in vitro evaluation model of feed mycotoxin reduction. Toxicol Vitro. 2017;38:108–16. doi: 10.1016/j.tiv.2016.10.003 WOS:000389167600013. 27737795

[42] Yu Y-H, Lai Y-H, Hsiao FS-H, Cheng Y-H. Effects of Deoxynivalenol and Mycotoxin Adsorbent Agents on Mitogen-Activated Protein Kinase Signaling Pathways and Inflammation-Associated Gene Expression in Porcine Intestinal Epithelial Cells. Toxins (Basel). 2021;13(5). 301 doi: 10.3390/toxins13050301 WOS:000654606100001. 33922863 PMC8146456

[43] Avantaggiato G, Solfrizzo M, Visconti A. Recent advances on the use of adsorbent materials for detoxification of Fusarium mycotoxins. Food Additives and Contaminants Part a-Chemistry Analysis Control Exposure & Risk Assessment. 2005;22(4):379–88. doi: 10.1080/19440049.2015.1125530 WOS:000229181700012. 16019808

[44] Holanda DM, Yiannikouris A, Kim SW. Investigation of the Efficacy of a Postbiotic Yeast Cell Wall-Based Blend on Newly-Weaned Pigs under a Dietary Challenge of Multiple Mycotoxins with Emphasis on Deoxynivalenol. Toxins (Basel). 2020;12(8). 504 doi: 10.3390/toxins12080504 WOS:000564104100001. 32781569 PMC7472238

[45] Van Le Thanh B, Lessard M, Chorfi Y, Guay F. The efficacy of anti-mycotoxin feed additives in preventing the adverse effects of wheat naturally contaminated with Fusarium mycotoxins on performance, intestinal barrier function and nutrient digestibility and retention in weanling pigs. Canadian Journal of Animal Science. 2015;95(2):197–209. doi: 10.4141/cjas-2014-126 WOS:000355743200008.

[46] Patience JF, Myers AJ, Ensley S, Jacobs BM, Madson D. Evaluation of two mycotoxin mitigation strategies in grow-finish swine diets containing corn dried distillers grains with solubles naturally contaminated with deoxynivalenol. Journal of Animal Science. 2014;92(2):620–6. doi: 10.2527/jas.2013-6238 WOS:000331106400023. 24398837

[47] Paulick M, Winkler J, Kersten S, Schatzmayr D, Frahm J, Kluess J, et al. Effects of oral exposure to sodium sulphite-treated deoxynivalenol (DON)-contaminated maize on performance and plasma concentrations of toxins and metabolites in piglets. Arch Anim Nutr. 2018;72(1):42–57. doi: 10.1080/1745039X.2017.1415550 WOS:000429416600003. 29271253

[48] Tran AT, Kluess J, Berk A, Paulick M, Frahm J, Schatzmayr D, et al. Detoxification of Fusarium-contaminated maize with sodium sulphite—in vivo efficacy with special emphasis on mycotoxin residues and piglet health. Arch Anim Nutr. 2018;72(1):58–75. doi: 10.1080/1745039X.2017.1418047 WOS:000429416600004. 29313386

[49] Anh-Tuan T, Kluess J, Berk A, Paulick M, Frahm J, Schatzmayr D, et al. Effects of a Fusarium Toxin-Contaminated Maize Treated with Sodium Sulfite on Male Piglets in the Presence of an LPS-Induced Acute Inflammation. Toxins (Basel). 2018;10(10). 419 doi: 10.3390/toxins10100419 WOS:000448820400040. 30340332 PMC6215154

[50] Mwaniki AW, Buis QR, Trott D, Huber L-A, Yang C, Kiarie EG. Comparative efficacy of commercially available deoxynivalenol detoxifying feed additives on growth performance, total tract digestibility of components, and physiological responses in nursery pigs fed diets formulated with naturally contaminated corn. Translational animal science. 2021;5(2):txab050–txab. doi: 10.1093/tas/txab050 MEDLINE:. 34085027 PMC8162626

[51] Guerre P. Worldwide Mycotoxins Exposure in Pig and Poultry Feed Formulations. Toxins (Basel). 2016;8(12). doi: 10.3390/toxins8120350 WOS:000389342000004. 27886128 PMC5198545

[52] Yiannikouris A, Poughon L, Francois J, Cameleyre X, Dussap GA, Bertin G, et al. Study of organic binders able to complex mycotoxins and limit their impact on animals and residues in edible animal products. Paris: Inst Natl Recherche Agronomique; 2002. 42- p.

[53] Nguyen-Ba H, Taghipoor M, van Milgen J. Modelling the feed intake response of growing pigs to diets contaminated with mycotoxins. Animal. 2020;14:S303–S12. Pii s175173112000083x doi: 10.1017/S175173112000083X WOS:323498313300011. 32349831 PMC7391214

[54] Wu L, Liao P, He L, Ren W, Yin J, Duan J, et al. Growth performance, serum biochemical profile, jejunal morphology, and the expression of nutrients transporter genes in deoxynivalenol (DON)-challenged growing pigs. Bmc Veterinary Research. 2015;11. 144 doi: 10.1186/s12917-015-0449-y WOS:000357307100001. PMC4490653 26138080

[55] Reddy KE, Song J, Lee H-J, Kim M, Kim D-W, Jung HJ, et al. Effects of High Levels of Deoxynivalenol and Zearalenone on Growth Performance, and Hematological and Immunological Parameters in Pigs. Toxins (Basel). 2018;10(3). 114 doi: 10.3390/toxins10030114 WOS:000428565500022. 29518941 PMC5869402

[56] Iordanov MS, Pribnow D, Magun JL, Dinh TH, Pearson JA, Chen SLY, et al. Ribotoxic stress response: Activation of the stress-activated protein kinase JNK1 by inhibitors of the peptidyl transferase reaction and by sequence-specific RNA damage to the alpha-sarcin/ricin loop in the 28S rRNA. Molecular and Cellular Biology. 1997;17(6):3373–81. doi: 10.1128/MCB.17.6.3373 WOS:A1997WZ63700042. 9154836 PMC232190

[57] Pestka JJ. Deoxynivalenol: Toxicity, mechanisms and animal health risks. Animal Feed Science and Technology. 2007;137(3–4):283–98. doi: 10.1016/j.anifeedsci.2007.06.006 WOS:000249473500006.

[58] Thanh BVL, Lemay M, Bastien A, Lapointe J, Lessard M, Chorfi Y, et al. The potential effects of antioxidant feed additives in mitigating the adverse effects of corn naturally contaminated with Fusarium mycotoxins on antioxidant systems in the intestinal mucosa, plasma, and liver in weaned pigs. Mycotoxin Research. 2016;32(2):99–116. doi: 10.1007/s12550-016-0245-y WOS:000374268300007. 27021614

[59] Juan-Garcia A, Carbone S, Ben-Mahmoud M, Sagratini G, Manes J. Beauvericin and ochratoxin A mycotoxins individually and combined in HepG2 cells alter lipid peroxidation, levels of reactive oxygen species and glutathione. Food and Chemical Toxicology. 2020;139. 111247 doi: 10.1016/j.fct.2020.111247 WOS:000526412300005. 32165234

[60] Oh SY, Mead PJ, Sharma BS, Quinton VM, Boermans HJ, Smith TK, et al. Effect of Penicillium mycotoxins on the cytokine gene expression, reactive oxygen species production, and phagocytosis of bovine macrophage (BoMacs) function. Toxicol Vitro. 2015;30(1):446–53. doi: 10.1016/j.tiv.2015.09.017 WOS:000367635500026. 26394380

[61] Horky P, Tmejova K, Kensova R, Cernei N, Kudr J, Ruttkay-Nedecky B, et al. Effect of Heat Stress on the Antioxidant Activity of Boar Ejaculate Revealed by Spectroscopic and Electrochemical Methods. Int J Electrochem Sci. 2015;10(8):6610–26. WOS:000359200400047.

[62] Gambacorta L, Olsen M, Solfrizzo M. Pig Urinary Concentration of Mycotoxins and Metabolites Reflects Regional Differences, Mycotoxin Intake and Feed Contaminations. Toxins (Basel). 2019;11(7). 378 doi: 10.3390/toxins11070378 WOS:000482110000053. 31262000 PMC6669694

[63] Winkler J, Kersten S, Valenta H, Huether L, Meyer U, Engelhardt U, et al. Simultaneous determination of zearalenone, deoxynivalenol and their metabolites in bovine urine as biomarkers of exposure. World Mycotoxin J. 2015;8(1):63–74. doi: 10.3920/wmj2014.1745 WOS:000349133200006.

[64] Rodriguez-Carrasco Y, Font G, Ruiz-Leal MJ, Houda B. A short study of deoxynivalenol correlation in diet and urine. Toxicol Lett. 2015;238(2):S66–S7. doi: 10.1016/j.toxlet.2015.08.235 WOS:000370693801073.

[65] Brezina U, Rempe I, Kersten S, Valenta H, Humpf HU, Danicke S. Diagnosis of intoxications of piglets fed with Fusarium toxin-contaminated maize by the analysis of mycotoxin residues in serum, liquor and urine with LC-MS/MS. Arch Anim Nutr. 2014;68(6):425–47. doi: 10.1080/1745039X.2014.973227 WOS:000344478400001. 25355041

[66] Bouchard MJ, Chorfi Y, Letourneau-Montminy MP, Guay F. Effects of deoxynivalenol and sodium meta-bisulphite on nutrient digestibility in growing pigs. Arch Anim Nutr. 2019;73(5):360–73. doi: 10.1080/1745039X.2019.1641369 WOS:000479459600001. 31342788

[67] Przybylska-Gornowicz B, Tarasiuk M, Lewczuk B, Prusik M, Ziolkowska N, Zielonka L, et al. The Effects of Low Doses of Two Fusarium Toxins, Zearalenone and Deoxynivalenol, on the Pig Jejunum. A Light and Electron Microscopic Study. Toxins (Basel). 2015;7(11):4684–705. doi: 10.3390/toxins7114684 WOS:000365647700020. 26569306 PMC4663528

[68] Gonkowski S, Gajecka M, Makowska K. Mycotoxins and the Enteric Nervous System. Toxins (Basel). 2020;12(7). 461 doi: 10.3390/toxins12070461 WOS:000557834200001. 32707706 PMC7404981

[69] Pierron A, Alassane-Kpembi I, Oswald IP. Impact of two mycotoxins deoxynivalenol and fumonisin on pig intestinal health. Porcine Health Manag. 2016;2. Unsp 21 doi: 10.1186/s40813-016-0041-2 WOS:000407481600001. 28405447 PMC5382503

[70] Kim SW, Holanda DM, Gao X, Park I, Yiannikouris A. Efficacy of a Yeast Cell Wall Extract to Mitigate the Effect of Naturally Co-Occurring Mycotoxins Contaminating Feed Ingredients Fed to Young Pigs: Impact on Gut Health, Microbiome, and Growth. Toxins (Basel). 2019;11(11). 633 doi: 10.3390/toxins11110633 WOS:000501604700044. 31683617 PMC6891535

[71] Zielonka L, Wisniewska M, Gajecka M, Obremski K, Gajecki M. Influence of low doses of deoxynivalenol on histopathology of selected organs of pigs. Polish Journal of Veterinary Sciences. 2009;12(1):89–95. WOS:000264277500013. 19459445

[72] Skiepko N, Przybylska-Gornowicz B, Gajecka M, Gajecki M, Lewczuk B. Effects of Deoxynivalenol and Zearalenone on the Histology and Ultrastructure of Pig Liver. Toxins (Basel). 2020;12(7). 463 doi: 10.3390/toxins12070463 WOS:326984271200001. PMC7404993 32698427

